# A Digital Mental Health Support Program for Depression and Anxiety in Populations With Attention-Deficit/Hyperactivity Disorder: Feasibility and Usability Study

**DOI:** 10.2196/48362

**Published:** 2023-10-11

**Authors:** Charalampos Tsirmpas, Maria Nikolakopoulou, Sharon Kaplow, Dimitrios Andrikopoulos, Panagiotis Fatouros, Konstantinos Kontoangelos, Charalabos Papageorgiou

**Affiliations:** 1 Feel Therapeutics Inc. San Francisco, CA United States; 2 First Department of Psychiatry Eginition Hospital Medical School National and Kapodistrian University of Athens Athens Greece; 3 Neurosciences and Precision Medicine Research Institute “Costas Stefanis” University Mental Health Athens Greece

**Keywords:** precision medicine, internet-based intervention, mobile apps, major depressive disorder, anxiety disorders, attention-deficit/hyperactivity disorder, personalized medicine, comorbidity, quality of life, mobile phone

## Abstract

**Background:**

A total of 1 in 2 adults with attention-deficit/hyperactivity disorder (ADHD) struggles with major depressive or anxiety disorders. The co-occurrence of these disorders adds to the complexity of finding utility in as well as adherence to a treatment option. Digital therapeutic solutions may present a promising alternative treatment option that could mitigate these challenges and alleviate symptoms.

**Objective:**

This study aims to investigate (1) the feasibility and acceptance of a digital mental health intervention, (2) participants’ engagement and retention levels, and (3) the potential efficacy with respect to anxiety and depression symptoms in a population with ADHD. Our main hypothesis was that a digital, data-driven, and personalized intervention for adults with coexisting ADHD and depressive or anxiety symptoms would show high engagement and adherence, which would be accompanied by a decrease in depressive and anxiety symptoms along with an increase in quality of life and life satisfaction levels.

**Methods:**

This real-world data, single-arm study included 30 adult participants with ADHD symptomatology and coexisting depressive or anxiety symptoms who joined a 16-week digital, data-driven mental health support program. This intervention is based on a combination of evidence-based approaches such as cognitive behavioral therapy, mindfulness, and positive psychology techniques. The targeted symptomatology was evaluated using the Patient Health Questionnaire–9, Generalized Anxiety Disorder–7, and Barkley Adult ADHD Rating Scale–IV. Quality of life aspects were evaluated using the Satisfaction With Life Scale and the Life Satisfaction Questionnaire, and user feedback surveys were used to assess user experience and acceptability.

**Results:**

The study retention rate was 97% (29/30), and high engagement levels were observed, as depicted by the 69 minutes spent on the app per week, 5 emotion logs per week, and 11.5 mental health actions per week. An average decrease of 46.2% (*P*<.001; *r*=0.89) in depressive symptoms and 46.4% (*P*<.001; *r*=0.86) in anxiety symptoms was observed, with clinically significant improvement for more than half (17/30, 57% and 18/30, 60%, respectively) of the participants. This was followed by an average increase of 23% (*P*<.001; *r*=0.78) and 20% (*P*=.003; *r*=0.8) in Satisfaction With Life Scale and Life Satisfaction Questionnaire scores, respectively. The overall participant satisfaction level was 4.3 out of 5.

**Conclusions:**

The findings support the feasibility, acceptability, and value of the examined digital program for adults with ADHD symptomatology to address the coexisting depressive or anxiety symptoms. However, controlled trials with larger sample sizes and more diverse participant profiles are required to provide further evidence of clinical efficacy.

## Introduction

### Background

Attention-deficit/hyperactivity disorder (ADHD), as defined in the Diagnostic and Statistical Manual of Mental Disorders, Fourth Edition (DSM-IV), is a heritable, chronic, and neurobehavioral disorder of childhood onset and is characterized by symptoms of inattentiveness and hyperactivity-impulsivity [1]. Although the exact etiology of ADHD is still unknown, there is an ever-growing body of neurobiological evidence that supports a multifaceted neurodiversity that contributes to impairment in multiple domains, such as cognitive processing, attention, motor planning, speed of processing responses, and other behavioral issues observed in ADHD [2]. Specifically, the prefrontal cortex, caudate, and cerebellum regions are interconnected by a network of neurons and together regulate attention, thoughts, emotions, behavior, and actions [1,3]. This neurobiological research adds a layer of insight into the presentation of ADHD symptoms, which begin in childhood, affecting the level of quality in social and occupational functioning, and continue to cause problems into adulthood for approximately 29% of people diagnosed [4]. The typical clinical picture of adults with ADHD is characterized by inattention or hyperfocus, hyperactivity, mind wandering, and impulsivity. Furthermore, emotional dysregulation and functional impairment are also characteristic features of adults with ADHD [1,2,5]. In terms of emotional dysregulation, the observed emotional changes are short-lived and usually triggered by daily activities [1-3,5], whereas functional deficits mainly include problems with inhibition and working memory; organizing, prioritizing, and initiating work; focusing, sustaining, and shifting attention to tasks; managing frustration; and self-regulating emotions and behaviors [1-3,5]. The array of symptoms and functioning in adults with ADHD suggests a multimodal treatment approach requiring a sophisticated treatment solution that can address the variety of symptoms.

The need to find a viable solution that can alleviate the burden of the varied symptoms of adults with ADHD, including the symptoms coming from coexisting disorders, is further emphasized by the worldwide prevalence of ADHD in the general adult population, which is approximately 2.5%. In addition, it is associated with substantial individual impacts such as difficulties in interpersonal functioning, educational and occupational performance, and community activities [6-9]. Patients with ADHD also have a high risk of criminal behavior and substance use disorders, an increased risk of accidents or unintentional injuries [10-13], and poor health and lifestyle choices [14-16]. Moreover, as many as 80% of patients with ADHD have at least 1 coexisting psychiatric disorder [6,17,18]. The most frequent comorbid psychopathologies include mood and anxiety disorders, bipolar disorder, and personality disorders. Approximately 20% to 50% of adults with ADHD have major depressive disorder (MDD) or dysthymia, whereas approximately 1 in 2 struggles with anxiety disorders (generalized anxiety disorder [GAD]) [17]. The co-occurrence of these disorders adds to the complexity of finding utility in as well as adherence to a treatment option. This highlights the need to find therapeutic solutions for the population with ADHD who have comorbid depression and anxiety disorders that will alleviate symptoms and positively affect their quality of life.

Traditionally, the treatment options for adults with ADHD are medication or psychotherapy as stand-alone treatments or in combination with each other. Although the standard of care for the comorbidity of MDD and GAD is similar to that of ADHD [17], the presence of these symptoms can mask symptoms of ADHD and forestall the diagnosis of ADHD in adults, which prevents the population from receiving appropriate treatment that addresses ADHD [3,5,17]. In addition, symptoms of depression and anxiety can create a barrier to the use of any relevant treatment option for ADHD [10,18,19]. An additional treatment factor to consider is that adults with ADHD tend to prefer nonpharmacological options for managing their symptoms [20,21]. Therefore, psychotherapy, which includes behavioral and cognitive behavioral therapy (CBT) both individually and in groups as well as skills training, can provide a suitable treatment option [22-24]. Finally, it is important to mention that the effectiveness of any intervention is closely related to keeping regular appointments and adherence to treatment recommendations [25,26]. Digital therapeutic solutions may present a promising alternative treatment option that could mitigate these challenges.

The scientific community has made great efforts to improve adherence and engagement with the introduction of tools such as digital mental health interventions. These digital tools stem from mobile health or digital health research to support behavior change, which has grown in relevance over the past 2 decades [27]. Digital interventions have the potential to evoke changes unavailable in traditional methods because of the ability to design personalized treatment models and measure engagement [28-30]. Increasing evidence focusing on the effectiveness of these interventions for MDD and GAD exists in the literature [31,32]. In addition, there is early evidence for adults with ADHD that shows positive clinical outcomes, suggesting that a digital solution can help improve co-occurring symptoms and daily functioning by providing digitally designed programs to meet the patient’s needs [1,20,21,33,34]. Furthermore, as many patients with ADHD prefer a nonpharmacological option to manage their symptoms [20,35], digital mental health interventions may offer a viable solution to meet their treatment needs. Patient adherence may also be improved by providing evidence-based solutions such as 24/7 access to educational information and resources, personalized support, and targeted interventions and skills training via technology (eg, apps or websites) [21,35] in the comfort of their own homes [20,30]. Digital mental health interventions are novel solutions, and consideration should also be given to newly developed industry guidelines and socioeconomic and cultural determinants that affect design and engagement [36-38]. Therefore, it is important to test the feasibility of new data-driven digital mental health interventions for adults with ADHD and comorbidities with the aim of improving their overall mental health status and quality of life.

In this study, we used a digital mental health solution that was designed and developed by Feel Therapeutics in the context of a real-world data, proof-of-concept study targeting a population with ADHD symptomatology and coexisting depressive or anxiety symptoms. The specific solution regards a digital, data-driven mental health support program that spans a 16-week period and combines emotion journaling, evidence-based CBT, mindfulness, and positive psychology techniques augmented with the Feel Digital Precision Medicine Platform. For this study, the main components of the program are (1) the Feel mobile app, which enables participants to journal their emotions, have access to other mental health–related metrics, and participate in the weekly sessions; (2) personalized weekly 15-minute coaching sessions; and (3) structured weekly mental health educational material and exercises. In addition, a wrist-worn device, the Feel Emotion Sensor, which continuously monitors changes in the participants’ autonomic nervous system and captures a series of mental health–related metrics, is provided as an optional component.

### Objectives

In this proof-of-concept study, we investigated (1) the feasibility and acceptance of a digital mental health program, (2) the participants’ engagement and retention levels during the study, and (3) the potential efficacy of the solution with respect to anxiety and depression symptoms in a specific population. Our main hypothesis was that a digital, data-driven solution customized to the individual would show high engagement and adherence levels, which is of utmost importance when considering the coexisting ADHD symptoms. In addition, we expected that this would be accompanied by a substantial decrease in depressive and anxiety symptoms along with an overall increase in quality of life and life satisfaction levels.

## Methods

### Study Design and Participant Recruitment

This was a prospective, decentralized, and single-arm feasibility study. Participants for this study were recruited via the following channels: (1) candidate referrals from the undergraduate student mental health support unit of the National and Kapodistrian University of Athens, (2) social media advertisements (eg, Facebook and Instagram), and (3) word of mouth. A custom web page, which included general information about the intervention and study, was built by Feel Therapeutics for study recruitment purposes. The study’s inclusion and exclusion criteria were evaluated based on self-reported candidate responses to the eligibility questionnaire, and the validity of their responses was also confirmed during their introductory session. The main inclusion criteria were (1) mild to severe MDD (Patient Health Questionnaire–9 [PHQ-9] ≥5) or GAD (Generalized Anxiety Disorder–7 [GAD-7] ≥5), (2) age of ≥18 years, and (3) smartphone ownership. In contrast, the main exclusion criteria were (1) personality disorders, (2) psychotic disorders, (3) bipolar disorder, (4) eating disorders, (5) suicidal or self-harm thoughts, (6) psychotropic medication, and (7) substance abuse.

Interested candidates could access the recruitment web page by either clicking on the social media advertisement or directly clicking on the web page link if recruitment took place through the National and Kapodistrian University of Athens or word of mouth. Therefore, the first step of the process was for interested candidates to visit the recruitment web page and complete the demographic and eligibility questionnaire, followed by the PHQ-9, GAD-7, Barkley Adult Attention-Deficit/Hyperactivity Disorder Rating Scale–IV (BAARS-IV), Life Satisfaction Questionnaire (LISAT-11), and Satisfaction With Life Scale (SWLS). Their responses were used to evaluate their eligibility for the study according to the study’s inclusion and exclusion criteria. The participant group that showed ADHD symptomatology (based on their responses to the BAARS-IV) as well as MDD or GAD symptoms (based on their responses to the PHQ-9 and GAD-7, respectively) was considered eligible for the study and was allocated to the 16-week intervention. For eligible candidates, these responses constituted the baseline values for the assessment of the progression of depressive and anxiety symptoms as well as quality of life and well-being levels. Candidates who did not fulfill the study’s inclusion criteria (regarding demographics and depression and anxiety symptoms; see the *Study Design and Participant Recruitment* section) were immediately disqualified and received proper communication.

**Figure 1:**
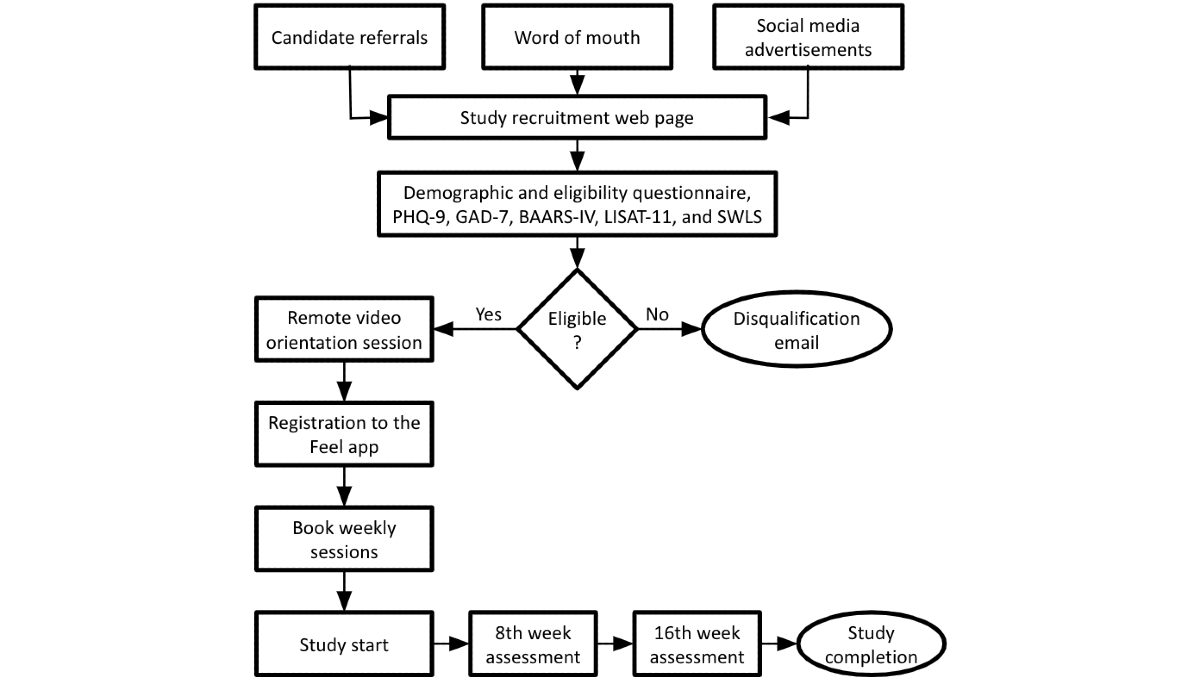
Overview of participant involvement steps. BAARS-IV: Barkley Adult Attention-Deficit/Hyperactivity Disorder Rating Scale–IV; GAD-7: Generalized Anxiety Disorder–7; LISAT-11: Life Satisfaction Questionnaire; PHQ-9: Patient Health Questionnaire–9; SWLS: Satisfaction With Life Scale.

**Figure 2:**
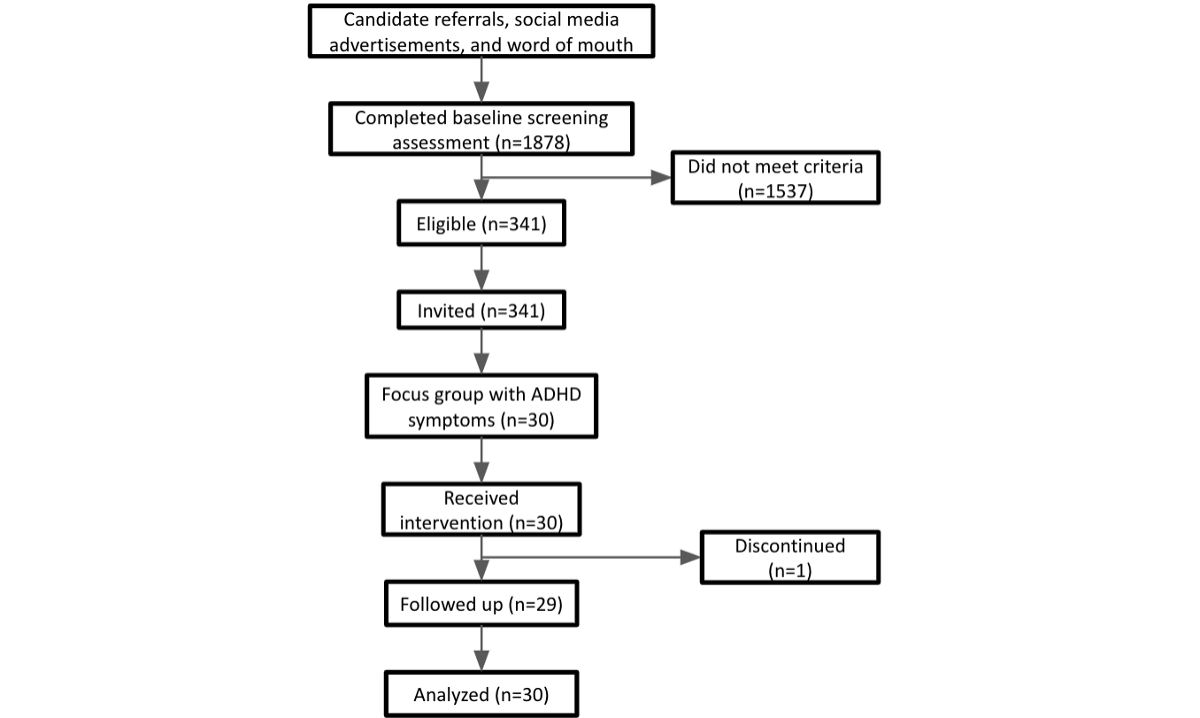
Participant funnel for the study. ADHD: attention-deficit/hyperactivity disorder.

Eligible candidates were invited to join the study, and a remote video orientation session was scheduled with a member of our customer success team in which they were informed about the study’s scope and components and became acquainted with the various features of the program. Moreover, the demographic information (eg, age and gender) collected in the demographic survey was cross-validated during this session. They then downloaded, installed, and registered to the Feel app and went through the app onboarding tasks that aimed to walk them through the different parts of the program. Among these, participants could check the providers’ availability, select their preferred one, and book their 16 weekly sessions. In addition, the (optional) Feel Emotion Sensor was shipped to the participants who opted to use it. Finally, the progression of their depressive and anxiety symptoms, along with their life satisfaction and quality of life levels, were monitored in the middle (ie, after the eighth session) and at the end of the study (ie, after the 16th session). The study took place between August 2022 and January 2023. A high-level presentation of the participant involvement step flow is illustrated in Figure 1, and the participant funnel for the study is presented in Figure 2.

### Materials

#### Demographic and Eligibility Questionnaire

This questionnaire was completed by candidates at the screening stage to assess their eligibility for the study and collect basic profiling information. The questions regarded basic demographic information (eg, gender, age, and location) as well as the presence of any of the inclusion and exclusion criteria. The demographic and eligibility questionnaire was embedded in the recruitment web page and completed at baseline.

#### PHQ-9 Scale

The PHQ-9 is a 9-item self-administered questionnaire widely used to assess the severity of depressive symptoms [39]. Each of the items composing the questionnaire scores the frequency of occurrence of the 9 DSM-IV criteria on a 4-point Likert scale from 0 (*not at all*) to 3 (*nearly every day*), with the overall score being the sum of the 9 individual scores. The total score serves as a classification threshold for the level of depressive symptoms. The PHQ-9 was completed at baseline (embedded in the recruitment web page) and at weeks 8 (midprogram) and 16 (end of program) on the Feel app.

#### GAD-7 Scale

The GAD-7 is a 7-item self-administered questionnaire that serves as a brief clinical measure for assessing the severity of GAD [40]. Each of the items composing the questionnaire scores the frequency of occurrence of symptoms on a 4-point Likert scale from 0 (*not at all*) to 3 (*nearly every day*), with the total score being the sum of the 7 individual scores. The total score serves as a classification threshold for the level of GAD symptoms. The GAD-7 was completed at baseline (embedded in the recruitment web page) and at weeks 8 (midprogram) and 16 (end of program) on the Feel app.

#### BAARS-IV Scale

The BAARS-IV is an 18-item self-administered questionnaire that is used to assess current ADHD symptoms and domains of impairment [41]. The item pool for the BAARS-IV consists of the 18 DSM-IV symptoms related to inattention, hyperactivity, and impulsivity rated on a 4-point Likert scale ranging from 1 (*never or rarely*) to 4 (*very often*). The frequency of current inattention or hyperactivity-impulsivity symptoms, along with the total ADHD score, are used for the evaluation of ADHD symptoms. The BAARS-IV was completed at baseline (embedded in the recruitment web page).

#### LISAT-11 Scale

The LISAT-11 is an 11-item self-administered questionnaire used for measuring life satisfaction [42]. The 11 items are used to evaluate life as a whole (1 global item) along with vocational and financial situation, leisure, contact with friends, sexual life, activities of daily living, family life, partnership relationship, physical health, and psychological health aspects (10 items). Each of the items is scored on a 6-point Likert scale ranging from 1 (*very dissatisfied*) to 6 (*very satisfied*), with the total score being the average of the 11 individual scores. The final score is used to evaluate quality of life and life satisfaction aspects. The LISAT-11 was completed at baseline (embedded in the recruitment web page) and at weeks 8 (midprogram) and 16 (end of program) on the Feel app.

#### SWLS Scale

The SWLS is a 5-item self-administered subjective well-being questionnaire that measures global life satisfaction [43]. Each item is scored on a 7-point Likert scale ranging from 1 (*strongly disagree*) to 7 (*strongly agree*), with the total score being the sum of the 5 individual scores. The final score is used to evaluate the global cognitive judgments of an individual’s life satisfaction. The SWLS was completed at baseline (embedded in the recruitment web page) and at weeks 8 (midprogram) and 16 (end of program) on the Feel app.

#### Self-Assessment Questionnaire

The self-assessment questionnaire is a custom 6-item self-administered questionnaire aiming to capture the participants’ perceptions regarding their accomplishments and progress throughout the 16 weeks of the study. Each item is scored on a 5-point Likert-type scale ranging from 1 (*strongly disagree*) to 5 (*strongly agree*). Indicative questions of this survey are “I learned one or more strategies to solve or cope with challenging situations” and “I have improved my lifestyle choices in at least one Wellness area (e.g. get more sleep, eat better, reach out to friends more, seek out time to connect with nature, etc.).” The self-assessment questionnaire was completed at week 16 (end of program) on the Feel app.

#### User Feedback Survey

The user feedback survey is a custom 12-item self-administered questionnaire used to evaluate various aspects related to the participants’ experience throughout the study, including overall level of satisfaction, as well as program component–specific feedback (eg, Feel mobile app, psychoeducational material, and customer support). The first 10 items, which are closed-ended, are scored on a 5-point Likert-type scale ranging from 1 (*not at all*) to 5 (*extremely*), whereas the last 2 items are open-ended. These allowed for open-ended comments about features of the program that the participants particularly liked or considered useful, as well as recommendations for improvements. The user feedback survey was completed after the end of the study on the Feel app.

#### Mobile App Interaction Metrics

A wide range of mobile app–related metrics were collected during the study, including participants’ emotion logs, completed exercises, number of attended weekly sessions, and overall time spent on the mobile app.

### Intervention: The Feel Program for Populations With ADHD

Feel Therapeutics has introduced an integrated digital mental health support program, the Feel Program for populations with ADHD, aiming to leverage precision medicine tools in the treatment of mental health disorders. These tools create an ecosystem that combines various evidence-based approaches, such as CBT, mindfulness, and positive psychology techniques, with numerous data modalities (eg, patient-reported outcomes, emotion journaling, and physiological data). These capture a diverse range of elements and factors that are strongly related to various mental health conditions (eg, mood level, social interactions, and symptom progression). The data-driven nature of the program is maximized when data collected from the Feel Emotion Sensor are available. The program that is currently available in the United States and Europe includes the ecosystem components outlined in Textbox 1.

**Feel Program for populations with attention-deficit/hyperactivity disorder (ADHD): ecosystem components.**

**Feel mobile app**

- The Feel mobile app serves as a digital front door to the Feel Program. This design creates a user-friendly environment for adults with ADHD and comorbidities. Each element of the app leverages behavioral activation principles to promote knowledge and awareness and build self-management skills. The user journey begins with the study onboarding step, which provides basic information about the scope and aims and walks participants through the value of the different program components. Participants can also schedule and attend their weekly personalized video sessions with a dedicated coach via the Feel app. In addition, the structured weekly mental health educational material and exercises are hosted on the app. Finally, the entirety of the patient-reported outcomes and surveys administered during the study are integrated into the app, and participants are notified to complete them at regular intervals. Finally, the Feel app collects the data from the Feel Emotion Sensor (FES) and transmits them to the Feel cloud-based processing infrastructure for the participants who opted to use the FES. The Feel mobile app is available on Android and iOS.

**Personalized weekly sessions**

- During the study, participants have access to 15-min remote weekly video sessions (apart from the introductory session that lasts 45 min) with their behavioral health coach. During the session, coaches leverage data provided by the Feel app and the FES along with a wide range of metrics via a dashboard to identify themes, keywords, and behavior patterns; connect the dots with the weekly material and exercises; and be able to target feedback and personalize interventions. Customizing the user experience has the potential to sustain attention and prompt the use of program modalities, which are typically behavioral challenges for adults with ADHD. In addition, the behavioral coping strategies and interventions are aligned with the transtheoretical model, also referred to as the stages of change model (ie, contemplation, preparation, action, maintenance, and relapse) [44,45], along with motivational interviewing in the weekly sessions.

**Structured weekly mental health educational material and exercises**

- This component is also embedded in the Feel mobile app and includes tutorials, exercises, and tools that focus on the challenges present when dealing with depression and anxiety. The 16-week clinical protocol comprises 3 phases. The first 3 weeks begin with identifying Specific, Measurable, Achievable, Relevant, and Time-Bound (SMART) goals [46] and familiarization with program components along with enhancing knowledge of the foundational theories and science behind the program interventions. The following 5 weeks build awareness of body-mind connection and implement cognitive restructuring, whereas the final weeks develop skills to increase resilience and self-management and implement long-term positive habits. The material is designed to keep the participants engaged during the study, support behavioral activation, and explain the rationale and scientific basis of the different interventions available during the study. These core interventions have been scientifically validated to support positive clinical outcomes [47]. The exercises complement the material and are customized by the coach based on the participants’ weekly data. Both the exercises and material accommodate the transtheoretical model. The digital delivery of this information enables audio, visual, and tactile stimulation and has the potential to captivate adults with ADHD and increase engagement.

**Feel Emotion Sensor (FES)**

- The FES is a wrist-worn device designed and manufactured by Feel Therapeutics that facilitates the ubiquitous monitoring of physiological data using 4 main sensors: an electrodermal activity sensor, a photoplethysmography sensor, a skin temperature sensor, and a 9-axis inertial measurement unit. Data collected by the FES are fused with data captured by the mobile device and the Feel app and uploaded to the Feel Digital Precision Medicine Platform (DPMP). The DPMP, a platform focusing on the monitoring and analysis of psychophysiological data, then extracts a variety of mental health–related metrics and markers, part of which are shared with the participants and their assigned behavioral health coach. The FES is an optional component for this study, but when chosen, it provides behavioral prompts to the user, which can be particularly helpful for a population of adults with ADHD.

### Emotion Journaling

The emotion journaling component, which is based on CBT principles, is a significant feature of the Feel Program as it enables participants to understand their thought, feeling, and behavior cycle; facilitates personalized in-the-moment interventions; and provides powerful insights to the coaches that are important for the weekly sessions. The journaling process can be initiated by either a notification sent by the Digital Precision Medicine Platform (when the Feel Emotion Sensor has been deployed) or by the participants themselves. In the former case, participants receive a distinct vibration pattern through their sensor followed by a notification on the Feel app and are requested to ideally confirm or reject (they can also skip if they are unsure) that they have experienced an emotional moment. Following the confirmation of an emotional moment, they are asked to select the exact emotion experienced among 4 different categories (positive or negative valence and low or high intensity), as well as further characterize it through a set of (predefined or custom) emotion tags that best describe it. These 2 mandatory steps are also the first steps of the user-triggered emotion journaling process. In both cases, participants are also asked to evaluate the perceived intensity of the experienced emotion on a scale of 1 to 10 (nonmandatory step). Participants are then given the option to add any triggers, thoughts, behaviors, and physical sensations that are related to the experienced emotion (nonmandatory step). The completion of all mandatory and nonmandatory steps constitutes an emotion journal, whereas only completing the mandatory steps is considered an emotion log. Finally, after successfully completing an emotion log or journal, participants are prompted to take an optional mood booster to support emotion regulation.

### Midprogram and End of Program Evaluations

The progression of participants’ depressive and anxiety symptoms was monitored immediately after the completion of the eighth week of the program along with their life satisfaction and well-being levels. The same questionnaires, followed by the self-assessment and user feedback surveys, were then completed at the end of the 16-week program. All surveys were administered via the Feel app.

### Ethical Considerations

This single-arm study was conducted in accordance with the Declaration of Helsinki and approved by the institutional review board of the Neurosciences and Precision Medicine Research Institute “Costas Stefanis” (approval 72/26072021). Before their participation in the study, all individuals were informed about the study scope, objectives, methodology, and components and provided written informed consent. Each participant was assigned a unique identifier after providing informed consent. The collected data were pseudonymized, and no personal identifiers were used during data processing and results reporting. Participants could withdraw from the study at any time. Finally, participants were not provided any monetary compensation for taking part in the study.

### Statistical Analysis

Regarding the assessment of the feasibility of the intervention, the overall onboarding process is summarized and presented, followed by the participant responses to the eligibility survey. For the rest of the analysis, an intention-to-treat approach was followed, and thus, all participants who took part in the intervention were considered. When assessing the participants’ level of engagement, the mean values and their respective SDs of a wide range of app-related metrics are presented. With respect to the preliminary assessment of the intervention effect on depressive and anxiety symptoms as well as on the participants’ quality of life and life satisfaction levels, average values at baseline, midprogram (ie, 8 weeks), and end of program (ie, 16 weeks) and their respective SEs were used. To compare the clinical and life satisfaction scores among the different evaluation periods, we first investigated the distributions of the paired score differences. Using Kolmogorov-Smirnov tests, we concluded that the score differences did not meet the criteria for normality. Consequently, as an alternative to the paired *t* test, which requires that the paired differences be normally distributed [48], we used the 2-tailed Wilcoxon signed rank test to investigate the statistical significance of our results. The specific test asserts whether the distribution of the differences is symmetrical around zero [49]. In addition, it is nonparametric and, thus, does not require any particular form for the underlying distributions that are examined. The cutoff *P* value for statistical significance was set at .05. For the effect size, we used the matched-pairs rank biserial correlation (*r*), which resembles the difference between the proportion of the data that, according to the statistical test, show favorable and unfavorable results [50]. For the PHQ-9 and GAD-7 questionnaires, a favorable result corresponded to a decrease in the respective score, whereas for the LISAT-11 and SWLS, it was associated with an increase in the score. Minimal clinically important differences were defined as at least 5 points for the PHQ-9 scores [51] and at least 4 points for the GAD-7 scores [52]. The missing values were assumed to be missing at random, accounting for 4% of the total assessment survey values used in the analysis, and thus, their impact was considered minimal. We imputed the missing values using the multivariate feature imputation available in the open-source *scikit-learn* Python package (Python Software Foundation) [53]. This method incorporates information from the entire feature set (ie, scores from all evaluation periods) to provide a better estimation of the missing values compared with simpler methods such as mean or median imputation [54]. Finally, regarding the self-assessment and user feedback questionnaires, all participants who successfully completed these were considered in the corresponding results. All participant data collected during the study were stored and processed in our secure cloud-based infrastructure in Europe. For privacy reasons and to adhere to the General Data Protection Regulation, all data were pseudonymized before any processing or insight extraction.

## Results

### Recruitment, Feasibility, and Acceptability

In total, 1878 individuals expressed their interest in participating in the study and completed the baseline screening assessment, of whom 341 (18.16%) were eligible to participate. Their responses to the eligibility questionnaire are presented in Table 1. It should be noted that the overall eligibility ratio was not directly derived as the combination of the individual ratios of each inclusion and exclusion criterion as most of the ineligible candidates failed to satisfy more than one criterion.

A focus group of 30 participants exhibiting ADHD symptomatology was identified and analyzed in this study. Their average age was 31.34 (SD 6.44) years, with a female-male distribution of 67% (20/30) to 33% (10/30). In addition, the vast majority of them (25/30, 83%) were either full-time employees or students, whereas more than half of them (17/30, 57%) held a bachelor’s degree and almost 1 in 4 (7/30, 23%) had a trade school degree. Moreover, their average baseline PHQ-9 and GAD-7 scores were 14.3 (SD 6.08) and 11.4 (SD 4.83), respectively, with an even distribution among different depression severity levels and a higher concentration at higher anxiety severity levels. Finally, their average baseline BAARS-IV score was 23.8 (SD 10.23). More information about the participants’ demographic characteristics and baseline assessment scores is presented in Table 2. Within this group, 97% (29/30) of the participants completed the 16 weeks of the study monitoring period, whereas 3% (1/30) discontinued the study for personal reasons.

**Table 1:** Candidate responses to the eligibility questionnaire (N=1878).

Candidate responses		Values, n (%)
**Bipolar disorder**		
	Yes	70 (3.72)
	No	1808 (96.27)
**Eating disorders**		
	Yes	351 (18.69)
	No	1527 (81.31)
**Psychotic disorders**		
	Yes	122 (6.5)
	No	1756 (93.5)
**Personality disorders**		
	Yes	133 (7.08)
	No	1745 (92.92)
**PTSD^a^**		
	Yes	227 (12.09)
	No	1651 (87.91)
**MDD^b^**		
	No	63 (3.35)
	Mild, moderate, moderately severe, or severe	1815 (96.65)
**GAD^c^**		
	No	137 (7.29)
	Mild, moderate, or severe	1741 (92.71)
**Substance abuse**		
	Yes	162 (8.63)
	No	1716 (91.37)
**Suicidal or self-harm thoughts**		
	Yes	291 (15.5)
	No	1587 (84.5)
**Psychotropic medications**		
	Yes	377 (20.07)
	No	1501 (79.93)
**Age (years)**		
	>18	16 (0.85)
	≤18	1862 (99.15)

^a^PTSD: posttraumatic stress disorder.

^b^MDD: major depressive disorder.

^c^GAD: generalized anxiety disorder.

**Table 2:** Participant demographic characteristics and baseline assessment scores (n=30).

Characteristic			Values, n (%)
**Age group (y)**			
	18-23	4 (13)	
	24-35	14 (47)	
	36-45	11 (37)	
	46-65	1 (3)	
**Sex**			
	Female	20 (67)	
	Male	10 (33)	
**Employment status**			
	Full time	19 (63)	
	Part time	2 (7)	
	Homemaker	1 (3)	
	Student	6 (20)	
	Casual	1 (3)	
	Unemployed	1 (3)	
**Level of education**			
	Secondary school	5 (17)	
	Bachelor’s degree	17 (57)	
	Master’s degree	1 (3)	
	Trade school	7 (23)	
**MDD^a^ symptom severity**			
	Minimal	1 (3)	
	Mild	8 (27)	
	Moderate	6 (20)	
	Moderately severe	7 (23)	
	Severe	8 (27)	
**GAD^b^ symptom severity**			
	Minimal	3 (10)	
	Mild	6 (20)	
	Moderate	11 (37)	
	Severe	10 (33)	

^a^MDD: major depressive disorder.

^b^GAD: generalized anxiety disorder.

Participant responses to the user feedback survey, which included questions addressing aspects such as participant satisfaction, mobile app usability, participant support services, and importance of program components followed by open-ended feedback, are presented in Table 3. For the closed-ended questions, a 5-point Likert-type scale was introduced, whereas for the open-ended questions, participants could suggest up to 3 features or components that they particularly liked and 3 that they would want to improve or add. The overall participant satisfaction level was 4.3 (SD 0.67) out of 5, with 87% (26/29) of the participants being very or extremely satisfied. Similarly, 86% (25/29) of them stated that the program met their expectations, whereas almost all (23/29, 79%) would recommend it to someone they knew (average score 4.8, SD 0.51 out of 5). Finally, 9 (26/29, 90%) out of 10 participants would be disappointed if they could no longer participate in the program. Regarding the usability of the Feel mobile app, 83% (24/29) of participants found it very easy to navigate through it, with 97% (28/29) of them being able to find the information they were looking for in the app. Participant support services were also rated very highly (4.7, SD 0.71 out of 5) as 93% (27/29) of participants had their questions or concerns addressed in a very responsive manner. Furthermore, when it comes to the importance of the different program components, the personalized weekly sessions received the highest score (4.9 out of 5), with an impressive 100% (29/29) of participants considering them very important, followed by the Mental Health Resource Center and Feel mobile app with average ratings of 4.7 and 4.3 out of 5, respectively. Finally, participants chose (1) cognitive restructuring and behavior modification exercises, (2) daily functioning support through weekly sessions, and (3) overall program structure and design as the program features that they liked the most. In contrast, the user experience with the notes sections in the exercises, access to technology-enabled material and personal data trends, and a more intuitive mobile app flow were identified as features that could be improved or added to the program.

**Table 3:** Participant responses to the user feedback survey (n=29).

Survey question and participant responses			Values, n (%)
**Participant satisfaction**			
	**Overall, how satisfied are you with the Feel Program?**		
		Extremely	13 (45)
		Very	13 (45)
		Neutral	3 (10)
		Slightly	0 (0)
		Not at all	0 (0)
	**How well did the Feel Program meet your expectations?**		
		Extremely	13 (45)
		Very	12 (41)
		Neutral	3 (10)
		Slightly	1 (3)
		Not at all	0 (0)
	**How likely are you to recommend the Feel Program to someone?**		
		Extremely	23 (79)
		Very	5 (17)
		Neutral	1 (3)
		Slightly	0 (0)
		Not at all	0 (0)
	**How would you feel if you had to give up the program?**		
		Very disappointed	11 (38)
		Somewhat disappointed	15 (52)
		Indifferent	1 (3)
		Somewhat relieved	2 (7)
		Very relieved	0 (0)
**Feel mobile app usability**			
	**How easy is it to navigate in the Feel app?**		
		Extremely	16 (55)
		Very	8 (28)
		Neutral	5 (17)
		Slightly	0 (0)
		Not at all	0 (0)
	**Were you able to find the information you were looking for on the Feel app?**		
		Extremely	17 (59)
		Very	11 (38)
		Neutral	1 (3)
		Slightly	0 (0)
		Not at all	0 (0)
**Participant support services**			
	**How responsive have we been to your questions or concerns about the Feel Program?**		
		Extremely	23 (79)
		Very	4 (14)
		Neutral	1 (3)
		Slightly	1 (3)
		Not at all	0 (0)
**Program component importance**			
	**Feel mobile app**		
		Extremely	17 (59)
		Very	7 (24)
		Neutral	4 (14)
		Slightly	0 (0)
		Not at all	1 (3)
	**Mental Health Resource Center**		
		Extremely	21 (72)
		Very	7 (24)
		Neutral	0 (0)
		Slightly	1 (3)
		Not at all	0 (0)
	**Personalized weekly sessions**		
		Extremely	26 (90)
		Very	3 (10)
		Neutral	0 (0)
		Slightly	0 (0)
		Not at all	0 (0)
**Open-ended feedback^a^**			
	**Features that participants particularly liked**		
		Cognitive restructuring and behavior modification exercises	19 (66)
		Daily functioning support through weekly sessions	16 (55)
		Program structure and design	9 (31)
	**Features to improve or add**		
		UX^b^ with notes in the exercises	7 (24)
		Technology-enabled material and personal data trends	5 (17)
		Mobile app flow	4 (14)

^a^Participants could suggest up to 3 features in each question, so the percentages do not add up to 100.

^b^UX: user experience.

### Participant Retention and Engagement

Of the group of 30 participants, 29 (97%) successfully completed the program and 1 (3%) decided to discontinue the program for personal reasons. Consequently, the overall retention rate during the 4-month study period was 97% (29/30). Focusing on overall participant engagement, 93% (28/30) of them were active on a weekly basis during the 16-week program for an average of 3.2 (SD 0.99) days per week. This translates to 69 (SD 18.04) minutes spent on the Feel mobile app on average every week, with participants accessing it approximately 20 (SD 6.72) times per week. Regarding emotion journaling, participants logged on average approximately 5 (SD 0.85) emotional events per week with an average journaling rate of 82.07%, whereas 52.51% (1318/2510) of the total logs concerned positive valence emotions and 47.49% (1192/2510) regarded emotions of negative valence. With respect to the personalized weekly sessions with the behavioral health coach, a 94.2% (452/480) session attendance was observed. At the same time, participants completed on average 3.48 (SD 0.79) mental health exercises per week. Aiming to capture the participants’ progress and their actions toward improving their mental health status (eg, emotion journaling and exercises), a new metric, the mental health actions, was introduced. Higher values of the mental health action metric indicated higher engagement and usability of mental health resources, and lower values indicated lower engagement and usability of mental health resources. For the focus group of this study, participants averaged 11.42 (SD 2.34) mental health actions per week.

### Preliminary Assessment of Impact on Depressive and Anxiety Symptoms

**Figure 3:**
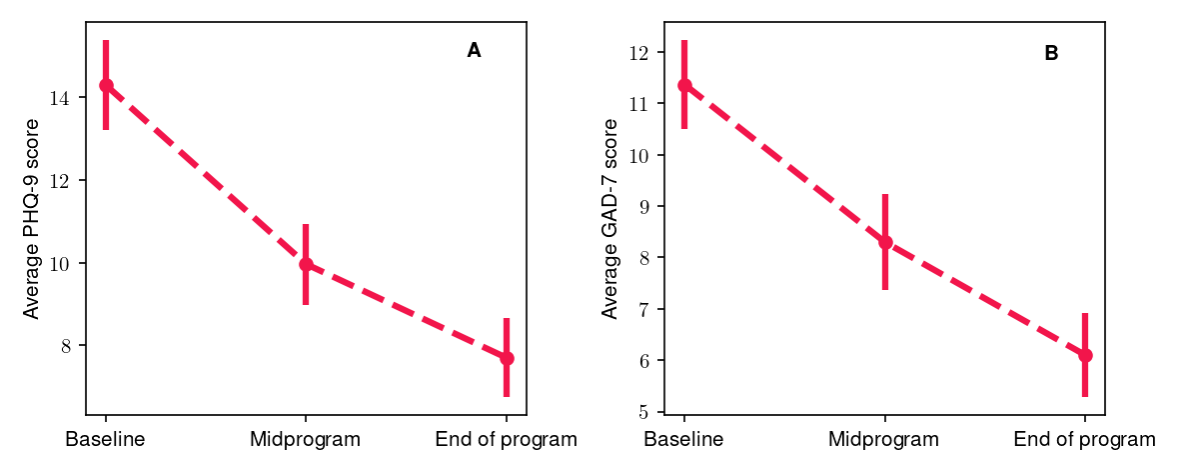
(A) Average participant Patient Health Questionnaire–9 (PHQ-9) and (B) average Generalized Anxiety Disorder–7 (GAD-7) scores at the baseline, midprogram, and end of program evaluations. The vertical bars represent the SE.

In Figure 3, we present the average values and their respective SEs for the participants’ PHQ-9 (Figure 3A) and GAD-7 (Figure 3B) scores at the baseline, midprogram, and end of program evaluations. Overall, a significant decrease was observed in both cases. More specifically, the average PHQ-9 score at baseline was 14.3 (SE 1.09), whereas at the midprogram and end of program evaluations, it decreased to 9.97 (SE 0.98) and 7.7 (SE 0.96), respectively. The relative baseline to midprogram and midprogram to end of program PHQ-9 score decrease was approximately 30.3% (*P*<.001; *r*=0.71) and 22.7% (*P*=.03; *r*=0.48), respectively, whereas the overall change (baseline to end of program) was 46.2% (*P*<.001; *r*=0.89). Similarly, the average GAD-7 score at baseline was 11.4 (SE 0.87), whereas at the midprogram and end of program evaluations, it decreased to 8.3 (SE 0.94) and 6.1 (SE 0.83), respectively. Pairwise comparisons among the different evaluation periods yielded a relative change of approximately 27% between the baseline and midprogram evaluations and between the midprogram and end of program evaluations. Both were statistically significant (*P*=.001 and *P*=.004, respectively) with medium to large effect sizes (*r*=0.74 for the former and *r*=0.68 for the latter). The overall change (baseline to end of program) was of the order of 46.3% (*P*<.001; *r*=0.86). All changes in average PHQ-9 and GAD-7 scores were statistically significant with medium to high effect sizes. More specifically, 87% (26/30) of the participants showed an improvement in depressive symptoms, and 77% (23/30) showed an improvement in anxiety symptoms. In addition, 77% (23/30) exhibited an improvement in both symptom categories, whereas 87% (26/30) showed an improvement in at least one of them. Finally, clinically significant improvement was observed in 57% (17/30) of the participants for depressive symptoms and in 60% (18/30) of the participants for anxiety symptoms. Overall, the statistical significance of the results suggests a potentially beneficial impact of the Feel Program on depression and anxiety symptoms.

### Preliminary Assessment of Impact on Quality of Life and Well-Being

**Figure 4:**
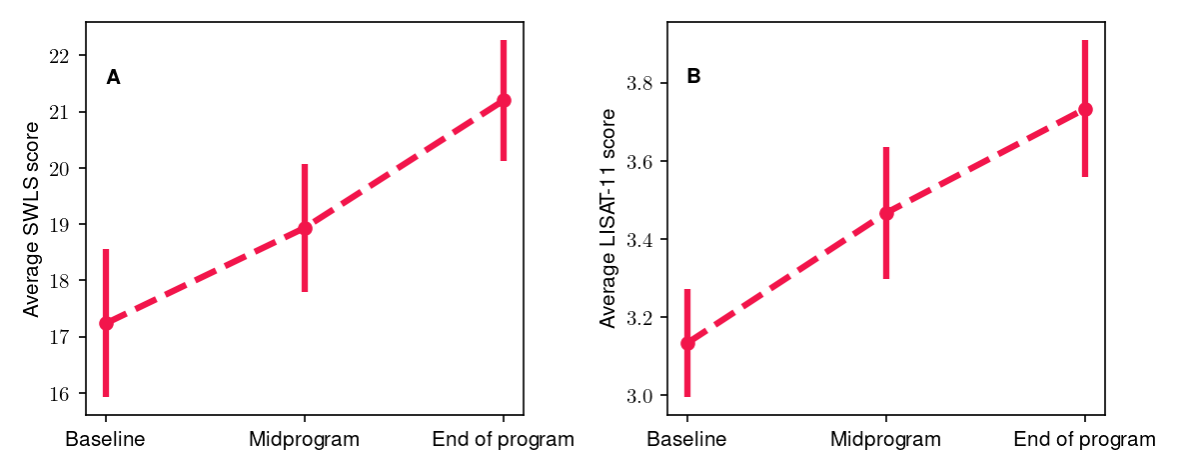
(A) Average participant Satisfaction With Life Scale (SWLS) and (B) average Life Satisfaction Questionnaire (LISAT-11) scores at the baseline, midprogram, and end of program evaluations. The vertical bars represent the SE.

Figure 4 shows the average values and their respective SEs for participants’ SWLS (Figure 4A) and LISAT-11 (Figure 4B) scores during the 3 evaluation periods. More specifically, for the SWLS, the average value at baseline was 17.2 (SE 1.32), whereas at midprogram it increased to 18.9 (SE 1.13), with a further increase to 21.2 (SE 1.08) at the end of program time point. Pairwise comparisons among the baseline, midprogram, and end of program evaluations yielded an average change of approximately 10% (*P*=.03; *r*=0.48) and 12% (*P*=.01; *r*=0.56), respectively, whereas the overall change (baseline to end of program) was 23% (*P*<.001; *r*=0.78). At the same time, for the LISAT-11, we observed an average value of 3.1 (SE 0.14) at baseline, whereas the corresponding value was 3.5 (SE 0.17) at midprogram and 3.7 (SE 0.18) for the end of program evaluation. The average increase between the baseline and midprogram evaluations and between the midprogram and end of program evaluations was 10.6% and 7.7%, respectively, which in both cases is characterized as marginally significant (*P*=.051 and *P*=.049, respectively). However, the overall change from the baseline to end of program assessment was 20%, which was statistically significant (*P*=.004; *r*=0.8). Thus, the overall change in participants’ SWLS and LISAT-11 scores was statistically significant with a large effect size. Finally, 67% (20/30) of participants exhibited an increase in SWLS scores, and 50% (15/30) exhibited an increase in LISAT-11 scores.

### Preliminary Assessment of Participant Self-Assessment Results

An additional tool aimed at evaluating the participants’ perceptions regarding their progress and learning during the program, the self-assessment survey, was introduced. Participants’ responses to the self-assessment survey are presented in Table 4, where it becomes evident that participants anticipated that significant progress would be achieved. In this context, 93% (27/29) of participants stated that they had learned strategies that would help them handle challenging situations, whereas almost all of them (19/29, 66%) learned coping strategies to reduce distressing emotions or behaviors. In addition, 2 out of 3 participants strongly believed that they had strengthened their self-management skills, with 86% (25/29) having improved their lifestyle choices (eg, getting more sleep and eating better). Finally, 83% (24/29) of the participants increased their ability to recognize and express their feelings, and 72% (21/29) of them found their self-confidence or self-esteem to be enhanced.

**Table 4:** Participant responses to the self-assessment questionnaire (n=29).

Survey question and participant responses			Values, n (%)
**I learned one or more strategies to solve or cope with challenging situations.**			
	Strongly agree	20 (69)	
	Agree	7 (24)	
	Neither disagree nor agree	2 (7)	
	Disagree	0 (0)	
	Strongly disagree	0 (0)	
**I learned to have balanced or alternative thinking as well as a growth mindset to reduce distressing emotions or behaviors.**			
	Strongly agree	19 (66)	
	Agree	9 (31)	
	Neither disagree nor agree	0 (0)	
	Disagree	1 (3)	
	Strongly disagree	0 (0)	
**I strengthened one or more self-management skills (eg, awareness of my thoughts, feelings, and behaviors; cultivating the positive; and self-care).**			
	Strongly agree	19 (66)	
	Agree	10 (34)	
	Neither disagree nor agree	0 (0)	
	Disagree	0 (0)	
	Strongly disagree	0 (0)	
**I have improved my lifestyle choices in at least one wellness area (eg, getting more sleep, eating better, reaching out to friends more, and seeking out time to connect with nature).**			
	Strongly agree	12 (41)	
	Agree	13 (45)	
	Neither disagree nor agree	4 (14)	
	Disagree	0 (0)	
	Strongly disagree	0 (0)	
**I increased my ability to recognize, name, and appropriately express my feelings.**			
	Strongly agree	16 (55)	
	Agree	8 (28)	
	Neither disagree nor agree	5 (17)	
	Disagree	0 (0)	
	Strongly disagree	0 (0)	
**I increased my self-confidence or self-esteem.**			
	Strongly agree	10 (34)	
	Agree	11 (38)	
	Neither disagree nor agree	5 (17)	
	Disagree	1 (3)	
	Strongly disagree	2 (7)	

## Discussion

### Principal Findings

This study was designed to explore and evaluate the feasibility and acceptance of a structured 16-week digital mental health program in a population with ADHD symptomatology, targeting co-occurring depression or anxiety symptoms. Specifically, this study focused on the utility of a digital mental health solution with this population; the opportunity for digitally designed and enhanced cognitive and behavioral interventions to positively affect engagement and adherence; and the potential relationship with the emergence of changes over time in psychometric indicators of depression, anxiety, and overall quality of life. The program may represent a valuable treatment solution as the population of adults with ADHD can be difficult to engage in any type of treatment because of the nature of their symptoms and functioning [25,26,55]. Adding to that the layer of a comorbidity such as depression or anxiety, it can become insurmountable [17,19,56] and challenge clinicians to find innovative ways of engagement and treatment delivery.

In this context, the main hypothesis of the study was that a data-driven, personalized, evidence-based digital therapeutic solution would support participant engagement along with enhanced exposure and adherence to clinical interventions, followed by an improvement in presenting symptoms. To support this hypothesis, three aims were investigated: (1) the feasibility and acceptance of a digital mental health program, (2) the participants’ engagement and retention levels during the study, and (3) the potential efficacy of the solution with respect to anxiety and depression symptoms in a specific population. The study results positively supported all 3 aims, as detailed in the following paragraphs. Highlights include high feasibility and acceptance of the solution based on the high interest in participating, completion rate of the onboarding steps, and very favorable scores on the user feedback survey. Moreover, we observed high participant retention and engagement, supported by the considerable time participants spent on the app and their overall activity per week. Finally, a substantial reduction in anxiety and depressive symptoms, followed by an improvement in quality-of-life measures, was reported by participants.

Program feasibility was evaluated based on 3 factors: the targeted population’s interest in the program and the operational and technical delivery of the program. First, a very high number of individuals (1878 candidates) expressed interest in participating in the study by completing the eligibility survey. The recruitment process yielded 341 eligible candidates who were interested in joining the study, of whom approximately 8.8% (30/341) self-reported ADHD symptomatology. Operational feasibility was then evaluated in the recruitment and onboarding phases. The study leveraged social media channels for recruitment, which created a digital front door for potential participants to be remotely assessed for eligibility. The onboarding workflow enabled participants to complete all digital onboarding steps on the same day. Finally, the program demonstrated technical feasibility through its availability via iOS or Android, which represent approximately 99% of mobile devices worldwide [57]. The Feel app enabled the delivery of digital evidence-based interventions, such as cognitive restructuring tools, emotion regulation techniques, and behavioral activation exercises. These interventions were accessible to the participants at any moment of need and leveraged digital behavior design elements to prompt behavior change and increase use. On the basis of these measures of feasibility, we consider the program to be a viable solution for the target population.

The acceptability of the program was assessed based on 3 different time points in relation to the intervention delivery period [58]. The time points were before intervention delivery, during intervention delivery, and after intervention delivery. To begin, all participants (30/30, 100%) attended the videoconference study orientation, whereas it is worth noting that 97% (29/30) of them also attended their introductory session. In addition, the vast majority (29/30, 97%) of participants who completed the in-app onboarding step attended their introductory session. Both of these preintervention delivery behaviors imply a perceived goodness of fit for the study and the program as the participants chose to continue on to the next step of the program. Interestingly enough, the participant who did not attend the introductory session eventually dropped out of the program. Next, the during-intervention-delivery behavior showed extremely high activity levels, with 93% (28/30) of participants using the app weekly for the duration of the program, accessing the app approximately 20 times per week. Finally, the postintervention delivery acceptability was rated through a user feedback survey. To begin, the overall participant satisfaction level was 4.3 out of 5, with 90% (26/29) of participants being very or extremely satisfied, along with 86% (25/29) of them stating that the program met their expectations. In further detail, 83% (24/29) of participants reported that the app was very or extremely easy to navigate, and a high percentage of participants saw the value in each of the program core components (Feel mobile app: 24/29, 83%; Mental Health Resource Center: 28/29, 97%; Personalized weekly sessions: 29/29, 100%; provided high or very high ratings). In addition, the survey results showed that 9 out of 10 participants would be disappointed if they could no longer take part in the program, whereas almost all (28/29, 97%) would recommend it to someone they knew. On the basis of this acceptance, we consider the Feel Program to be highly tolerable for this patient population.

The favorable acceptability of the program was represented by complimentary participant engagement and retention, which are a particularly important aspect for this population as treatment adherence can be challenging. The study retention rate was 97% (29/30), with participants being engaged on an average of 3.2 days per week. With respect to the different study components, participants were actively engaged. More specifically, they spent an average of 69 minutes on the mobile app weekly with a 94.2% (452/480) session attendance, whereas they frequently used the journaling component, with an average of 5 emotional events per week and a journal completion rate of 82.11% (2061/2510). The mental health exercises were observed to be an important component as well, with participants averaging 3.48 exercises per week. A high use rate was observed for the “Express gratitude,” “Body scan,” and “Daily activities” exercises, which address common symptoms that persist in this study population [59]. The overall high engagement of the participants can be summarized through our mental health action metric, which attained a weekly average of 11.42.

As we further explore the current impact of digital mental health interventions, we recognize that, although there is recent research on the application of digital therapeutic models in adults with ADHD, to the best of our knowledge, there is no evidence that focuses on the co-occurrence of MDD and GAD symptomatology in adults with ADHD. Therefore, this study offers a preliminary look into the possibility of using such digital interventions for this population. For depression and anxiety symptoms as indicated by PHQ-9 and GAD-7 scores, respectively, a continuing decrease in average scores was observed from baseline to midprogram and all the way to the end of the program. The overall PHQ-9 and GAD-7 change (baseline to end of program) was approximately 46% and statistically significant for both scales, suggesting that there was overall progress on both scales. This progress is reflected in improvements in the symptoms as reported through participants’ responses. We also observed clinically significant changes in 57% (17/30) of participants for depressive symptoms and in 60% (18/30) for anxiety symptoms. This may be related to the high level of participant engagement and adherence to the personalized, data-driven program components. The program’s personalized direction (eg, emotion journaling, exercises, and weekly sessions) helped participants attain their goals, such as developing emotion regulation, strengthening resilience, and building self-management skills. By achieving their weekly goals, they were able to redefine their beliefs about themselves, contributing to changes in their depression and anxiety symptoms. The effectiveness of the program’s interventions aligns with previous research on this population that suggests that cognitive restructuring is a helpful tool to address self-limiting thinking and recalibrate beliefs about what behaviors can affect positive change [60-65]. Participants in this study were able to move from intention to action by promoting tasks of engagement rather than avoidance [60-65]. Overall, the statistical significance of the results suggests a potentially beneficial impact of the FP on depression and anxiety symptoms.

As we continue to explore the progression of depression and anxiety symptoms, we consider the potential connection between the use of the transtheoretical model and motivational interviewing to promote behavioral activation and positively affect health outcomes. In this study, participants were either at the contemplation stage (20/30, 67%) or the preparation stage (10/30, 33%), which was evaluated by the behavioral health coach during the introductory session. During the study, the 2 groups showed different engagement behaviors. More specifically, participants at the preparation stage had a mean BAARS-IV score of 26.4 (SE 3.37), spent on average 1.42 hours on the mobile app, and averaged 16.8 mental health actions on a weekly basis. At the same time, participants at the contemplation stage had a mean score of 22.5 (SE 2.23) on the BAARS-IV questionnaire, spent approximately 1 hour on the mobile app, and completed on average 8.7 mental health actions per week. Nevertheless, a significant observational result was that both groups of participants showed significant reductions in depression and anxiety symptoms. In more detail, we observed an approximately 50% overall reduction in PHQ-9 and GAD-7 scores (*P*<.001; *r*=0.98 in both cases) in the contemplation stage group, whereas for the preparation stage group, the overall reduction was 41% (*P*=.04; *r*=0.73) in PHQ-9 scores and 42% (*P*=.14; *r*=0.61) in GAD-7 scores. We also observed that 77% (23/30) of participants showed improvement in depression and anxiety symptomatology regardless of their stage of change (contemplation or preparation).

Finally, improvements in symptoms were followed by a subsequent increase in health-related quality of life metrics (baseline to end of program evaluations), as reflected by participants’ responses to the SWLS and LISAT-11. These measures are of particular relevance to the patient population, which faces a serious disruption to life domains [8-10,14]. In summary, this study suggests that the investigated 16-week digital mental health intervention (ie, Feel Program for populations with ADHD) for a population of adults with ADHD who have MDD and GAD symptoms retains high levels of participant engagement following clinically significant reductions in MDD and GAD symptoms and overall improvement in quality of life and life satisfaction.

### Limitations

This study has several limitations that may affect the generalizability of the results and should be considered when interpreting them. The most important among these is the sample size of the study, which was relatively small, with 97% (29/30) of the participants successfully completing the study. In addition, all recruited participants had self-reported symptoms of ADHD and MDD or GAD. Considering the small sample size and the lack of a professional diagnosis, we are unable to generalize the findings to a broader population with ADHD experiencing depression and anxiety symptoms. Furthermore, the lack of a control group significantly restrains our ability to support the effectiveness of the intervention and associate it with overall depressive and anxiety symptom changes and improvements in quality of life. Finally, we did not monitor the participants’ level of proficiency with mobile devices, and thus, assessing its impact on participants’ engagement was not possible. Overall, this study suggests that an investment in future studies that address these limitations and strengthen the integrity of the outcomes to apply the findings to adults with ADHD and co-occurring symptoms of depression and anxiety would be beneficial.

Considering the aforementioned aspects, future studies should include a broader and more diverse sample population to validate the outcomes of this study and mitigate potential bias, along with a professional medical diagnosis that will validate the participant profiles. To this end, various enhancements to the recruitment process, such as engaging community clinics along with ADHD groups, experts, and foundations, could be very important. In addition, the inclusion of a control group would provide strong evidence regarding the efficacy of the intervention. It would also be valuable to explore the impact of access to the digital solution and monitor any constraints because of smartphone ownership, data use, and technical proficiency. In addition, we believe that it would be very interesting to explore the potential effect of the observed improvement in depressive and anxiety symptoms on ADHD symptomatology treatment. Finally, comparison groups to determine the impact of medication or alternative treatments could also be considered.

## Abbreviations

ADHD: attention-deficit/hyperactivity disorder
BAARS-IV: Barkley Adult Attention-Deficit/Hyperactivity Disorder Rating Scale–IV
CBT: cognitive behavioral therapy
DSM-IV: Diagnostic and Statistical Manual of Mental Disorders, Fourth Edition
GAD: generalized anxiety disorder
GAD-7: Generalized Anxiety Disorder–7
LISAT-11: Life Satisfaction Questionnaire
MDD: major depressive disorder
PHQ-9: Patient Health Questionnaire–9
SWLS: Satisfaction With Life Scale

## Data Availability

The original contributions presented in this study are included in the paper; further inquiries can be directed to the corresponding authors.
